# The Molecular Network behind Volatile Aroma Formation in Pear (*Pyrus* spp. Panguxiang) Revealed by Transcriptome Profiling via Fatty Acid Metabolic Pathways

**DOI:** 10.3390/life12101494

**Published:** 2022-09-26

**Authors:** Huiyun Li, Jine Quan, Sohel Rana, Yanmei Wang, Zhi Li, Qifei Cai, Shuhong Ma, Xiaodong Geng, Zhen Liu

**Affiliations:** 1 College of Forestry, Henan Agricultural University, Zhengzhou 450002, China; 2 Ying Lin Station Anyang Forestry Bureau, Anyang 455000, China

**Keywords:** pears, volatile aroma formation, fatty acid pathway, RNA-Seq, WGCNA

## Abstract

**Simple Summary:**

Pear is a widely eaten fruit all over the world. Volatile aroma is an important factor affecting fruit quality and the fatty acid metabolism pathway is important in synthesizing volatile aromas. In this study, Panguxiang (*Pyrus* spp. Panguxiang) is a new variety bred from *P*. *bretschneideri* Rehd. cv. ‘Biyang piaoli’ and, unlike most white pear varieties cultivated in China, its aroma is also vital. The study aimed to explore unique pear resources of rich fruit aroma and to clarify the metabolism and regulation mechanism of the aromatic components in pear fruit. This paper used physiological and transcriptome methods to explore the molecular network behind volatile aroma formation in Panguxiang revealed via fatty acid metabolic pathways. Through transcriptome sequencing, weighted gene co-expression network analysis (WGCNA) identified yellow functional modules and several biological and metabolic pathways related to fatty acid formation. Finally, we identified seven and eight hub genes in the fatty acid synthesis and fatty acid metabolism pathways, respectively. Further analysis of the co-expression network allowed us to identify several key transcription factors related to the volatile aroma, including AP2/ERF-ERF, C3H, MYB, NAC, C2H2, GRAS, and Trihelix, which may also be involved in fatty acid synthesis and further influence the formation of aroma.

**Abstract:**

Pears are popular table fruits, grown and consumed worldwide for their excellent color, aroma, and taste. Volatile aroma is an important factor affecting fruit quality, and the fatty acid metabolism pathway is important in synthesizing volatile aromas. Most of the white pear varieties cultivated in China are not strongly scented, which significantly affects their overall quality. Panguxiang is a white pear cultivar, but its aroma has unique components and is strong. The study of the mechanisms by which aroma is formed in Panguxiang is, therefore, essential to improving the quality of the fruit. The study analyzed physiological and transcriptome factors to reveal the molecular network behind volatile aroma formation in Panguxiang. The samples of Panguxiang fruit were collected in two (fruit development at 60, 90, 120, and 147 days, and fruit storage at 0, 7, 14, 21, and 28 days) periods. A total of nine sample stages were used for RNA extraction and paired-end sequencing. In addition, RNA quantification and qualification, library preparation and sequencing, data analysis and gene annotation, gene co-expression network analysis, and validation of DEGs through quantitative real-time PCR (qRT-;PCR) were performed in this study. The WGCNA identified yellow functional modules and several biological and metabolic pathways related to fatty acid formation. Finally, we identified seven and eight hub genes in the fatty acid synthesis and fatty acid metabolism pathways, respectively. Further analysis of the co-expression network allowed us to identify several key transcription factors related to the volatile aroma, including AP2/ERF-ERF, C3H, MYB, NAC, C2H2, GRAS, and Trihelix, which may also be involved in the fatty acid synthesis. This study lays a theoretical foundation for studying volatile compounds in pear fruits and provides a theoretical basis for related research in other fruits.

## 1. Introduction

Aroma is an important indicator of fruit quality [1], and rich fruit aromas can alter trends in consumption, improving human standards of living and increasing demand for fruits. The study of fruit aroma is therefore receiving increasing attention [2].

The types and proportions of the molecular components of fruit aroma can differ significantly between species, and there are also differences between different varieties of the same species. Pears are a vital fruit globally, with white pears (*Pyrus bretschneideri*) being the most widely cultivated varieties in China, therefore being often referred to as “Chinese pears”. Panguxiang (*Pyrus* spp. Panguxiang) is a new variety bred from *P. bretschneideri* Rehd. cv. ‘Biyang piaoli’. Panguxiang is highly nutritious and tastes sweet, crisp, and slightly sour. The aroma has unique components, but unlike most white pear varieties cultivated in China, the aroma is also strong. Due to scattered operations and poor management by farmers, the quality of Panguxiang has gradually decreased. Therefore, it is of great significance to study the anabolic mechanism behind the aroma of Panguxiang and effectively promote the regulation and accumulation of volatile substances.

Research into the volatile substances produced by pears began in 1964 [3]. Since then, there have been many studies on the characteristic volatiles of different pear species or varieties [4,5], including on the synthesis of aromatic compounds [6] and on the influence of ripening [7] and storage [8] stages on the aroma. Researchers have detected more than 300 volatile substances released by pear fruits, including aldehydes, alcohols, esters, terpenes, hydrocarbons, and sulfur-containing compounds [9]. Of these compounds, volatile esters and alcohols are known to be the most significant contributors to the aroma of certain fruits, including apple [10], strawberry [11], and banana [12]. A previous study reported that fatty acid metabolism is a major pathway through which volatiles (particularly esters) are formed in pears following the incubation of fruits with linoleic acid (LA, C18:2) and linolenic acid (LNA, C18:3) [13].

Straight-chain aliphatic alcohols, aldehydes, ketones, and esters are mainly generated via the fatty acid metabolic pathway [14]. In this pathway, the synthesis of volatile substances begins with C18 organic compounds, such as unsaturated fatty acids. The LA and LNA can generate straight-chain aldehydes, alcohols, and esters through the lipoxygenase (LOX) enzymatic system, which comprises four key enzymes, including LOX, hydroperoxide lyase (HPL), alcohol dehydrogenase (ADH), and alcohol acyltransferase (AAT) [15]. After LA and LNA enter the LOX pathway, they first form two intermediates—9-hydroperoxide and 13-hydroperoxide—under the catalysis of LOX, which are then further metabolized through the LOX pathway [16]. Next, these two hydroperoxide fatty acid derivatives are converted into C6 and C9 aldehydes through the action of HPL. These aldehydes are reduced to alcohols under the action of ADH and are further converted into esters under the action of AAT [14]. The ester compounds catalyzed by AAT are responsible for the strong aromas of fruits.

There have been many studies on volatile compounds in different pear varieties [5,17,18,19,20]. However, limited research has concentrated on the mechanism of volatile compound synthesis in Panguxiang. The current study focuses on the changes in volatile substance synthesis during the development and storage of Panguxiang fruits. In addition, the molecular regulatory network of volatile substance synthesis during fruit ripening and storage was analyzed with the WGCNA package. The results lay a theoretical foundation for the mechanism of fruit aroma synthesis and provide a theoretical basis for improving Panguxiang aroma.

## 2. Materials and Methods

### 2.1. Plant Materials and Sample Preparation

This experiment was carried out in the Key Laboratory of Forest Resources Cultivation of Henan Agricultural University and the State Forestry Administration (112°42′114°14′ E, and 34°16′34°58′ N). The soil condition at the experimental site was sandy loam with a pH value of 7.0. The study was conducted in 2021. A 6-year-old Panguxiang pear tree with strong growth was selected as the test tree. Thirty days after full flowering (May 2021), the middle and upper parts of the fruit tree in the four cardinal directions without diseases and pests were selected, and uniform fruits were produced for bagging.

To analyze the variations of the parameters in different stages (growth and development and storage period), a total of nine stages were selected to sample the fruits. Four stages of pear fruit growth and development were sampled: 60 days (S1), 90 days (S2), 120 days (S3), and 147 days (S4) after anthesis. When the fruits on the tree were ripe, samples of similar size were picked and stored in an artificial incubator (temperature 20 °C, humidity 70%) as samples for the storage period. Five stages of pear fruit storage were sampled: 0 days (Z1), 7 days (Z2), 14 days (Z3), 21 days (Z4), and 28 days (Z5) of storage. After sampling, all samples were stored in a −80 °C refrigerator. All samples at the nine stages were used for the RNA extraction and paired-end sequencing (samples from each period were collected in triplicate).

### 2.2. Determination of Volatile Substances

The samples taken at the nine different stages were removed from the −80 °C storage freezer and separately ground in liquid nitrogen. The samples taken at each stage were evenly divided into three 15 g replicates, and a total of 27 samples were used to determine the content of volatile substances. The specific test method follows that described by Fan et al. [21].

### 2.3. RNA Quantification and Qualification

Total RNA was isolated and purified using the CTAB method [22]. The integrity, purity, and concentration of the purified RNA were assessed using an Agilent 2100 Bioanalyzer and a NanoDrop ND-1000 spectrophotometer (NanoDrop Technologies, Wilmington, DE, USA). The mRNA extracted from the total RNA in the samples was isolated using Oligo dT.

### 2.4. Library Preparation and Sequencing

Libraries were generated and purified using the NEBNext^®^ Ultra™ RNA Library Prep Kit for Illumina^®^ (New England Biolabs Inc., Ipswich, MA, USA) and AMPure XP Beads (Beckman Coulter, Inc., Indianapolis, IN, USA), using the fragmented mRNA as the template and following the manufacturer’s recommendations. The concentration, integrity, and quantification of the library were determined by using a Qubit™ Fluorometer (ThermoFisher Scientific, Waltham, MA, USA), the KAPA Library Quantification Kit (KAPA Biosystems, Wilmington, MA, USA), and a Qsep100 DNA Analyzer (KAPA Biosystems), respectively. The denatured libraries were subjected to high-throughput parallel sequencing of both ends of the library using an Illumina HiSeq X™ Ten System sequencing platform. The quality of the raw data was evaluated using default settings.

### 2.5. Data Analysis and Gene Annotation

The raw data were filtered through fastp v0.19.3 [23] to obtain clean data. HISAT v2.1.0 [24] was used to map clean reads to the reference genome. The transcripts were quantified using featureCounts v1.6.2 [25], and the lengths of the transcripts in the sample were normalized to FPKM (fragments per kilobase of exon per million fragments mapped) values. The differential gene expression was analyzed between different groups using DESeq2 v1.22.1 [26]. After the difference analysis, the *p* value was corrected using the Benjamini and Hochberg method to obtain the false discovery rate (FDR). Genes with an expression-level change of |log2 (fold change)| ≥ 1 and FDR < 0.05 were considered to be differentially expressed genes (DEGs). The transcript sequences were mapped to seven public databases: the NCBI non-redundant protein sequences database (NR) (https://www.ncbi.nlm.nih.gov/ accessed on 25 August 2022) [27]; Swiss-Prot [28,29]; Gene Ontology (GO) [30]; euKaryotic Ortholog Groups (KOG) [31]; Protein family (Pfam) [32]; Kyoto Encyclopedia of Genes and Genomes (KEGG) [33]; and TrEMBL [34]. Mapping was conducted using BLAST software (E-value ≤ 10^−5^) to obtain annotation information for the transcripts [35]. The protein sequences were submitted to iTAK [36] to identify the transcription factors.

### 2.6. Gene Co-Expression Network Analysis

All the identified DEGs were used to construct a co-expression network using the R package WGCNA [37]. The co-expression modules were obtained using the automatic network construction function (blockwiseModules) with power = 18 and minModuleSize = 60. The TOM type was unsigned and the modules with highly correlated eigengenes (correlation > 0.85) were merged. The eigenvalue was calculated for each module based on Pearson correlation. The networks were visualized by Cytoscape [38].

### 2.7. Validation of DEGs through Quantitative Real-Time PCR (qRT–PCR)

Total RNA extracted from the fruit of Panguxiang was reverse transcribed using the FastQuant RT Kit with DNase (TianGen Biotech Co., Ltd., Beijing, China) to synthesize first-strand cDNA. A qRT–PCR assay was performed with an optical 96-well reaction plate, the ABI PRISM 7500 Real-time PCR system (Applied Biosystems, Foster City, CA, USA), and SuperReal PreMix Plus SYBR Green (TianGen Biotech Co., Ltd.). Each reaction contained 12.5 µL SYBR Premix ExTaq, 0.5 µL ROX reference dye, 2.0 µL cDNA, and 1.0 µL gene-specific primers in a final volume of 25 µL. All the primers were at concentrations of 10 μΜ. The primer sequence of genes is presented in Table 1. The PCR program was as follows: 95 °C for 10 s and then 45 cycles at 95 °C for 5 s and 60 °C for 40 s; tubulin was used as a reference. The qRT–PCR data were analyzed using the 2^−∆∆CT^ method [39]. The RNA concentration ranged from 600 ng/μL to 800 ng/μL, and the A260/A280 value ranged from 1.8 to 2.0. The initial concentration of cDNA was 1 μg/μL. The cDNA was diluted to four concentrations: 10^0^, 10^−1^, 10^−2^, and 10^−3^ (1 μg/μL). The E-value (amplification efficiency) of the qRT–PCR was between 90% and 110%, and R^2^ was greater than 0.99. The qRT–PCR of each gene was carried out three times to give three experimental replicates, and each experiment comprised three biological replicates [40].

## 3. Results

### 3.1. Sampling and Determination of Volatile Substance Content

**Figure 1:**
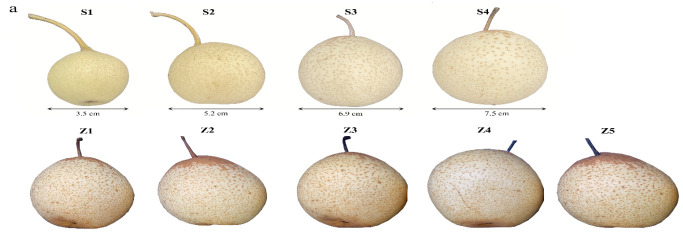

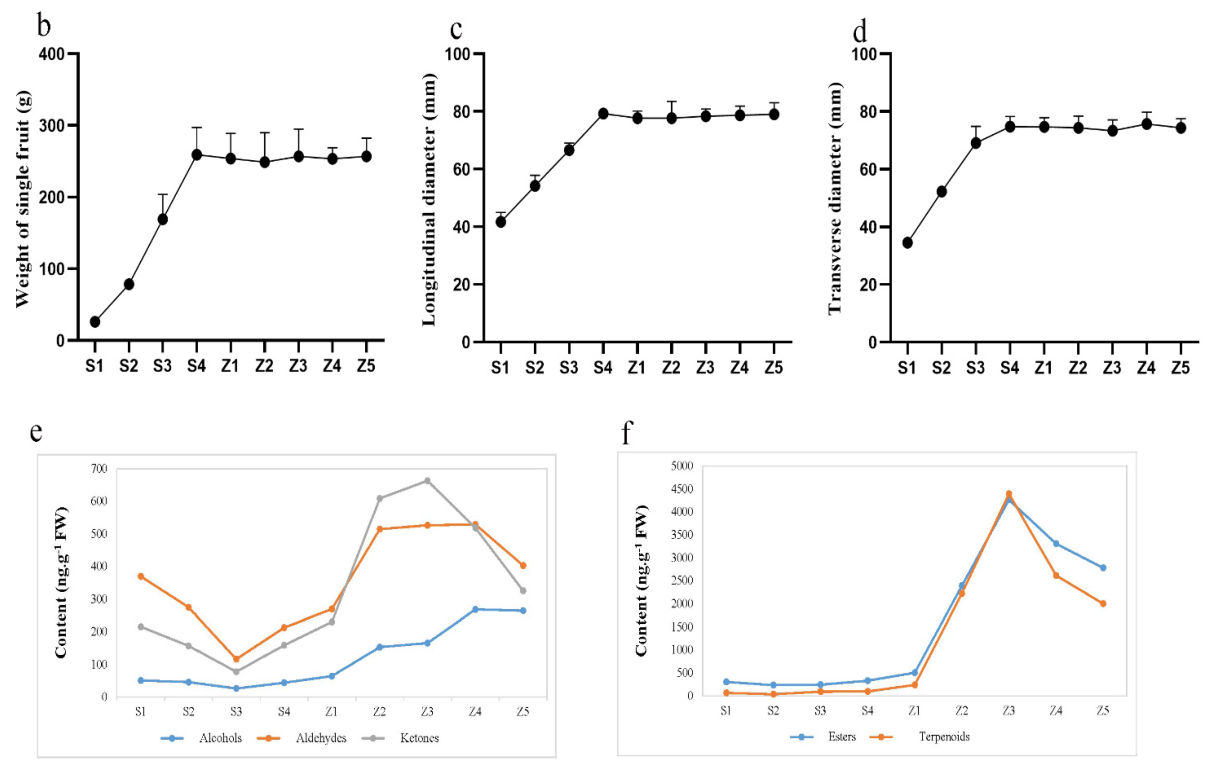
Phenotypic analysis of Panguxiang in different ripening and storage stages. (**a**) The phenotype of Panguxiang over the nine studied stages. (**b**–**d**) Visualized phenotypic analysis of ripening fruits. (**e**,**f**) Determination of concentrations of volatile compound groups in fruit over the nine studied stages.

We measured the fruit weight and size at the time of sampling. With the ripening of the fruits, their weight and size also increased, with the weight at stage S4 reaching 260 g. (Figure 1a–d). To analyze the aroma components of Panguxiang fruits at different stages, we determined the volatile substances present in the nine stages of the fruits. These volatiles included alcohols, ketones, aldehydes, esters, and terpenes. The levels of alcohols, ketones, and aldehydes in the nine stages initially decreased with ripening, but increased significantly starting at stage S3. The concentrations of aldehydes and ketones continued to increase throughout the first storage stages, reaching maxima at Z3 and Z4, after which they decreased once more. In contrast, the concentrations of alcohols, although lower than those of the other chemical groups, continued to rise throughout storage. The concentrations of esters and terpenoids remained unchanged throughout the ripening stages, but increased sharply during the first three stages of storage to a maximum at stage Z3 (4265 ng. g^−1^ and 4394 ng. g^−1^, respectively), after which they decreased again. At the early stage of storage, from Z1 to Z3, fruit esters and terpenoids rose rapidly; at the later stage of storage, the decrease rate of ester concentrations was slightly slower than that of terpenoid concentrations (Figure 1e,f).

### 3.2. Transcriptomic Analysis of Pyrus spp. Panguxiang Fruits

This study investigated the transcriptomic sequences of 27 samples taken at different fruit ripening and storage stages. A total of 183.32 GB of clean data was obtained. The clean data from each sample reached 6 GB and the Q30 score was >91% in each case (Table S1). The clean read sequences were aligned to the reference genome using HISAT2. The overall alignment ratio of each sample was higher than 70% (Table S2). The FPKM distribution of the transcriptome sequence data was visualized with box plots and violin plots to compare the overall transcript expression levels in the different samples. Gene expression was stable between the 27 samples (Figure S1a,b). Principal component analysis (PCA) and Pearson correlation coefficient (PCC) confirmed that the transcriptome characteristics were highly correlated between the biological replicates of each group of samples (Figure S1c,d).

A BLAST search was conducted to explore the functions of the unigenes and obtain annotation information for the transcripts. Functional annotations were performed using multiple public databases, including Nr, KEGG, GO, SwissProt, KOG, TrEMBL, and Pfam. The numbers of transcripts annotated by the seven databases were as follows: 39,707 in Nr, 34,069 in GO, 29,399 in KEGG, 24,306 in KOG, 33,220 in Pfam, 29,449 in SwissProt, and 40,424 in TrEMBL (Table S3). In identifying the DEGs in the nine different study stages, RNA sequencing (RNA-Seq) was performed to profile the dynamic changes in genome-wide transcript abundance and pairwise DESeq2 analysis between different time points was carried out. Hierarchical cluster analysis was then conducted according to the FPKM expression data after the standardization of differential genes, and cluster heatmaps were drawn (Figure S1e). We found large and significant differences in the number of DEGs among the nine study stages. In the comparative analysis of DEGs in different storage periods, the number of DEGs decreased from Z2/Z1 4382 (2638 upregulated and 1744 downregulated) to the subsequent period Z3/Z2 1184 (635 upregulated and 549 downregulated), respectively (Table S4). There were 1137 DEGs in Z4/Z3 (770 upregulated and 367 downregulated) and 1917 DEGs in Z5/Z4 (1285 upregulated and 632 downregulated). In the early stage of fruit storage, most DEGs were found in Z2/Z1. It is also possible that the transcriptional expression level of genes precedes the phenotypic changes observed (Figure S1f, Table S2).

### 3.3. Functional Annotation and Classification of DEGs

We analyzed the GO, KOG, and KEGG pathways to elucidate the biological functions of DEGs. The GO annotation system consists of three major branches: “biological process”, “molecular function”, and “cellular component”. These unigenes were further divided into 49 to 57 major functional terms. The “cellular process”, “cell part”, and “binding” were the most over-represented terms in the three GO categories mentioned above (Figure S2). The unigenes enriched by KOG could be assigned to 25 groups. Group R (general function prediction only) was the most highly represented. Groups T (signal transduction mechanisms) and O (posttranslational modification, protein turnover, chaperones) also shared a high percentage of genes. Only a few genes were assigned to Groups Y (nuclear structure), W (extracellular structures), and N (cell motility) (Figure S3). The “fatty acid metabolism pathway” was also indicated in the KEGG signaling pathway (Figure S4).

### 3.4. Co-Expression Analysis Identifies Key Gene Modules Involved in the Formation of Aroma Volatiles

To fully understand the correlation of gene expression during the formation of aroma volatiles, all DEGs were used in WGCNA and 15 co-expressed gene modules were identified (Figure S5). Module size ranged from 87 to 11,092 genes, with black (721 genes), blue (3377 genes), brown (3111 genes), cyan (136 genes), green (1519 genes), green-yellow (350 genes), grey (287), magenta (625 genes), midnight blue (87 genes), pink (662 genes), purple (613 genes), red (827 genes), salmon (263 genes), tan (346 genes), yellow (1953 genes), and turquoise (11,092 genes) represented.

The gene expression profile was visualized with the eigengene values for each module and showed distinct co-expression patterns across modules (Figure S5). The expression trend of the yellow and purple modules is consistent with the changing trend of aroma components in pear fruit. None of the other 13 modules were correlated with any variable. These results suggest that the yellow and purple modules may be the key to understanding the mechanism of pear aroma formation. However, the network formed by key genes in the purple module was less extensive, so we focused here on the analysis of the yellow module.

Transcription factors (TFs) are important regulators in plant development [41], and we therefore investigated the distribution of TF genes in all modules (Table S5). Statistical analysis suggested that the prominent TF families present in the yellow module were the AP2/ERF-ERF, C3H, MYB, NAC, C2H2, GRAS, and Trihelix families (Table 2, Table S6).

### 3.5. Co-Expression Network Analysis

**Figure 2:**
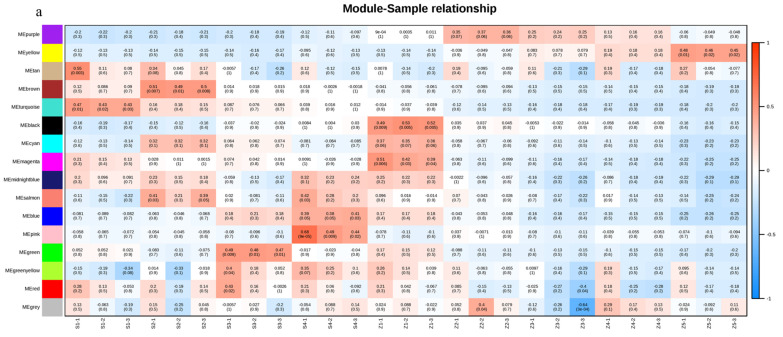

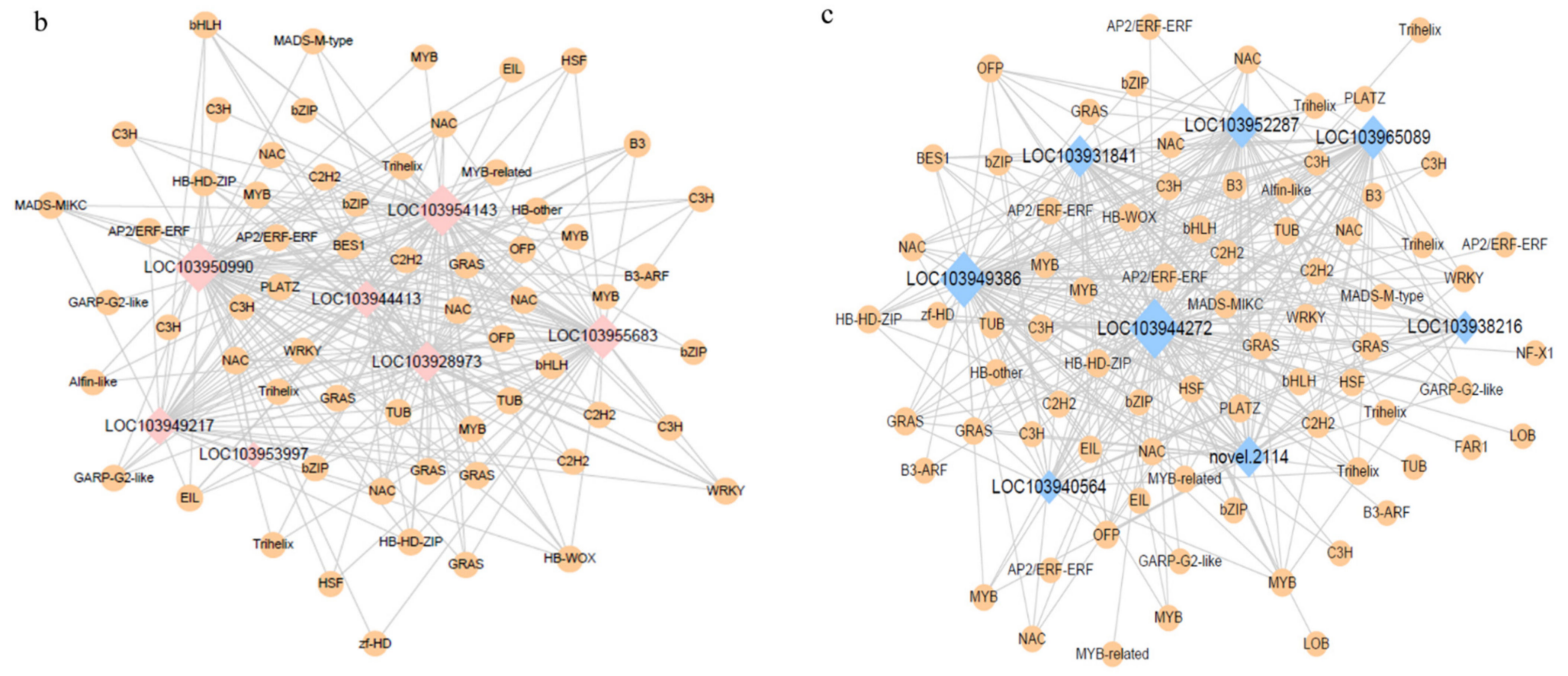
The co-expressed gene modules were identified by weighted gene co-expression network analysis (WGCNA). (**a**) Heatmap of correlations of expressed genes between samples and modules. (**b**) Co-expression network of the fatty acid synthesis pathway in the yellow module. (**c**) Co-expression network of the fatty acid metabolism pathway in the yellow module. For emphasis purposes, the circles representing hub genes are artificially enlarged.

To explore the correlations between each module and the samples taken at different stages, we drew a heatmap illustrating the correlations between the samples and the modules. The yellow and purple modules had the strongest correlations with later storage stages (Figure 2a), which is consistent with the previous result. The co-expression network analysis showed correlations between gene expression patterns and we constructed a network of detected co-expressed modules to identify key hub genes. A few hub genes interact with many other genes in gene networks. In this study, we analyzed the co-expression networks of the fatty acid synthesis pathway and the fatty acid metabolism pathway in the yellow module. We identified seven and eight key hub genes, respectively. The important regulatory genes involved in the fatty acid synthesis pathway were identified in those from the alcohol dehydrogenase (ADH 1, lysophospholipid acyltransferase LOC103931841; ADH 2, phospholipase A1LOC103965089; ADH 3, alcohol dehydrogenase (NADP+)LOC103949386; ADH 4, shikimate O-hydroxycinnamoyltransferase LOC103944272; ADH 5, 1-acyl-sn-glycerol-3-phosphate acyltransferaseLOC103938216; and ADH 6, palmitoyltransferase LOC103940564) and alcohol acyltransferase (AAT; alcohol dehydrogenase (NADP+) LOC103952287) genes, and the important regulatory genes involved in the fatty acid metabolism pathway were identified (acetyl-CoA carboxylase, biotin carboxylase subunit LOC103928973; 3-oxoacyl-[acyl-carrier protein] reductaseLOC103944413; long-chain acyl-CoA synthetaseLOC103949217; acetyl-CoA carboxylase biotin carboxyl carrier proteinLOC103950990; enoyl-[acyl-carrier protein] reductaseLOC103953997; long-chain acyl-CoA synthetaseLOC103954143; acetyl-CoA carboxylase biotin carboxyl carrier protein LOC103955683, and novel.2114 (glycerol-3-phosphate acyltransferase)). (Figure 2b,c).

### 3.6. Verification of the Expression Profiles of Key Genes

**Figure 3:**
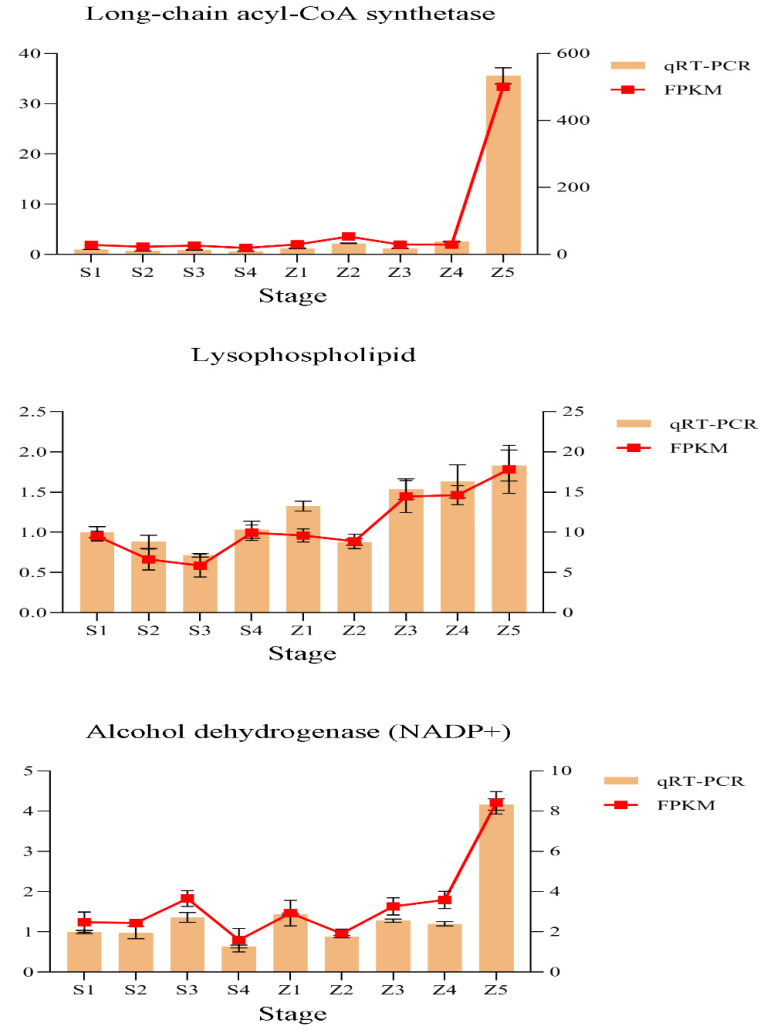
Verification of the expression profiles of representative genes from different modules using quantitative real-time PCR. Relative gene expression levels were calculated using tubulin as the internal reference. The results are presented as the means ± SE, and each sample represents three biological replicates.

To verify the RNA-Seq data, we used qRT–PCR to analyze the expression levels of three genes involved in the modules from the fatty acid synthesis and metabolism pathways. The gene expression patterns were then compared with the FPKM values. The results showed that the expression patterns suggested by the two analysis techniques were consistent, indicating that our data were reliable (Figure 3 and Table 1).

## 4. Discussion

### 4.1. Effect of Fruit Ripening and Storage on Aroma Content

The synthesis of fruit aroma is a dynamic process and most substances important for fragrance appear in the later stage of fruit development, stimulated by the release of ethylene [42]. Of the different classes of volatile chemicals, research suggests that esters and aldehydes are the most abundant in pears, regardless of species or cultivar [6,43]. Unripe fruits mainly contain “green flavor” aroma substances such as aldehydes and alcohols, with aldehyde and alcohol concentrations gradually decreasing and ester concentrations gradually increasing as the fruits mature [44]. As the most abundant class of volatiles, esters contribute ‘sweet’ and ‘fruity’ notes to the aroma of different fruits [45]. This is consistent with our findings. In our study, the size and weight of the fruits increased as they matured, and the concentrations of alcohols, ketones, and aldehydes decreased. At the same time, ester concentrations tended to remain constant, which may result from accumulation. Esters were significantly upregulated when the fruits were fully ripe, with the upregulation lasting until the Z3 stage. The concentrations of volatile aroma components showed a similar trend during storage, increasing first and then decreasing, but during the storage stages, the esters and terpenoids were at much higher levels than the aldehydes and alcohols. It is possible that the fruits continued to ripen, even after picking, until stage Z3. At stage Z3, the volatile aroma content peaked, after which the fruits were affected by the low storage temperature and the concentrations of volatile aroma components began to decrease. In general, the main volatile aromas produced by Panguxiang during fruit growth and development are alcohols and aldehydes, while the main volatile aromas released during storage are esters and terpenoids.

### 4.2. WGCNA Explores the Formation of Volatile Aromas in Pear Fruits

WGCNA is a widely used data mining method. Exploration of the genes whose expression patterns closely correlate with each other can be achieved through the application of WGCNA [46]. Although WGCNA can be applied to most high-dimensional data sets, it is most widely used in genomes, especially transcriptomes [47]. It allows the definition of a module’s network nodes, hub, and membership, as well as the study of the relationship between the co-expressed modules and the comparison of the topologies of different networks [37]. Through this algorithm, the genes that are co-expressed with high correlation are placed in one module, and different modules may contain key genes implicated in the volatile aroma formation process [48].

Our results showed that the purple and yellow modules had the strongest correlation with the S3, S4, and S5 stages, and samples taken at the later storage stages also had the highest volatile aroma content. Based on our choice, the yellow module was also an essential factor. In the yellow module, we analyzed the co-expression networks of two pathways: the fatty acid synthesis pathway and the fatty acid metabolism pathway. We identified seven and eight key hub genes from these two pathways.

### 4.3. Key Factors in the Formation of Volatile Aroma in Pears

It should be noted that the biosynthesis of esters via the LOX pathway is largely dependent on an adequate supply of substrate [49]. Aldehydes are reduced to alcohols by ADH [50], and fruit esters are then biosynthesized by AAT, with the expression of AAT being ethylene-dependent in climacteric fruits such as apple [51] and pear [6]. In our study, ADH genes (LOC103931841, LOC103965089, LOC103949386, LOC103944272, LOC103938216, LOC103940564, and novel.2114) and AAT genes (LOC103952287) in the fatty acid metabolism pathway were identified by co-expression network analysis. A large amount of ethylene is produced during the maturation and storage of Panguxiang. The AAT gene is induced by ethylene and regulates the production of volatile esters, resulting in the development of a rich fruit flavor during ripening and storage. Pears have a “green” aroma when they are not ripe, which is caused by volatile alcohol. ADH genes are the key genes in the regulation of volatile alcohol production. Our research shows that alcohol concentrations are higher when fruits are riper and stored for longer, which may result from the accumulation of ADH, providing further evidence that ADH and AAT genes play an important role in forming volatile aromas.

## Figures and Tables

**Table 1:** The primer sequence of genes.

Gene Name	Gene ID	Primer Sequence (5′-3′)		The Size Products
long-chain acyl-CoA synthetase	LOC103949217	F:AACCGACTATCTTCTGTGCTGTTCC	R:AGCCGCCTGATGAAATCTTCTGTG	81 bp
lysophospholipid acyltransferase	LOC103931841	F:CCAATGCTACTCGGCTATGCTTCC	R:CGATTCCTCCTTCCTTCCATGCG	139 bp
alcohol dehydrogenase (NADP+)	LOC103952287	F:CACCAGCATTGGGCTTGAAATCG	R:CACCTCCATCCCTGCCTCCTTC	121 bp
endogenous reference gene	AY338250	F:CAGGCTGACTGTGCTGTCCT	R:TCACACCGAGGGTGAAAGCA	115 bp

**Table 2:** The number and types of TFs in different modules.

TF Family	Black	Blue	Brown	Cyan	Green	Green-Yellow	Grey	Magenta	Midnight Blue	Pink	Purple	Red	Salmon	Tan	Yellow	Turquoise
AP2/ERF-ERF		21	7	1	10					10			2		8	38
C2H2	1	4	8		5			2		3	2	6		1	5	32
C3H		13	9	1	10		2	2		2		5		1	7	22
GRAS		4	10		10										5	26
MYB	1		7	1	7		1	4		3	2	2			7	37
NAC	2	4	11		8				1	4	2				6	36
Trihelix		2	1		4			1		2					6	22
Total	4	48	53	3	54	0	3	9	1	24	6	13	2	2	44	213

## Data Availability

Not applicable.

## Supplementary Materials

The following supporting information can be downloaded at: https://www.mdpi.com/article/10.3390/life12101494/s1, Figure S1: RNA-Seq analysis of Panguxiang over different growth and development stages and storage stages. (**a**,**b**) Boxplots and violin plots of gene expression levels. (**c**) Principal component analysis and (**d**) sample correlation based on the RNA-Seq count matrix. (**e**) Heatmap of DEGs. (**f**) Numbers of differentially expressed genes in different pairs of samples. Figure S2: GO analysis diagram of growth and development period and storage period. Figure S3: KOG-enriched analysis of growth and development period and storage period. Figure S4: Analysis of KEGG pathway enrichment, growth and storage period. Figure S5: 15 co-expressed gene modules of growth and development period and storage period. Table S1: Data preprocessing results statistics. Table S2: The results of the genomic comparison. Table S3: The number of differentially expressed genes. Table S4: Summary of functional annotation. Table S5: The number of TFs in different modules. Table S6: The number and types of TFs in different modules.
