# Supplementary figures and images for: The Molecular Network behind Volatile Aroma Formation in Pear (Pyrus spp. Panguxiang) Revealed by Transcriptome Profiling via Fatty Acid Metabolic Pathways
Source: Life (Basel). 2022 Sep 26;12(10):1494. doi: 10.3390/life12101494 (PMC9605550; doi:10.3390/life12101494)

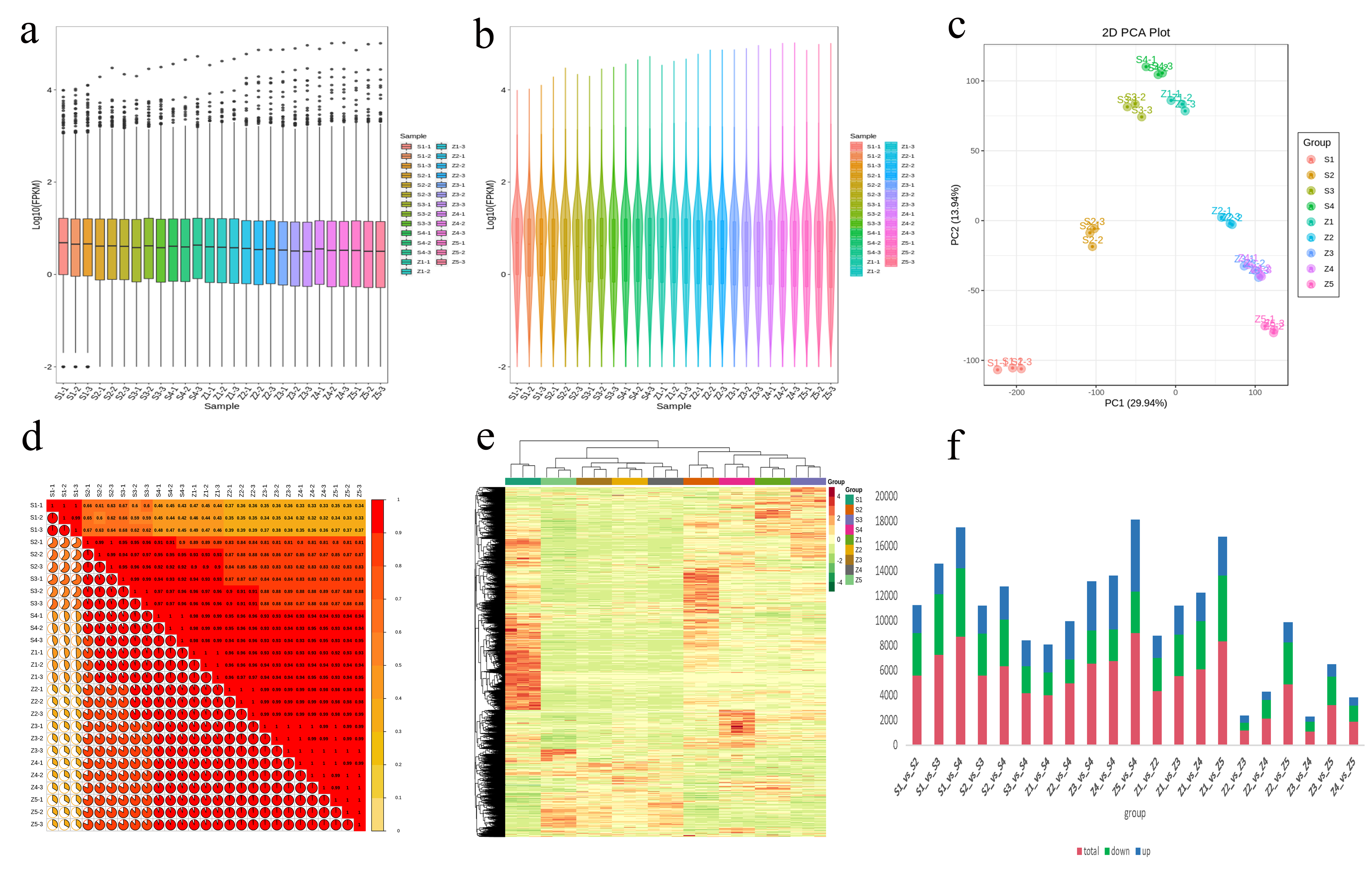

Supplement: Supplementary file 1 [file life-12-01494-s001.zip › Figure S1.tif]

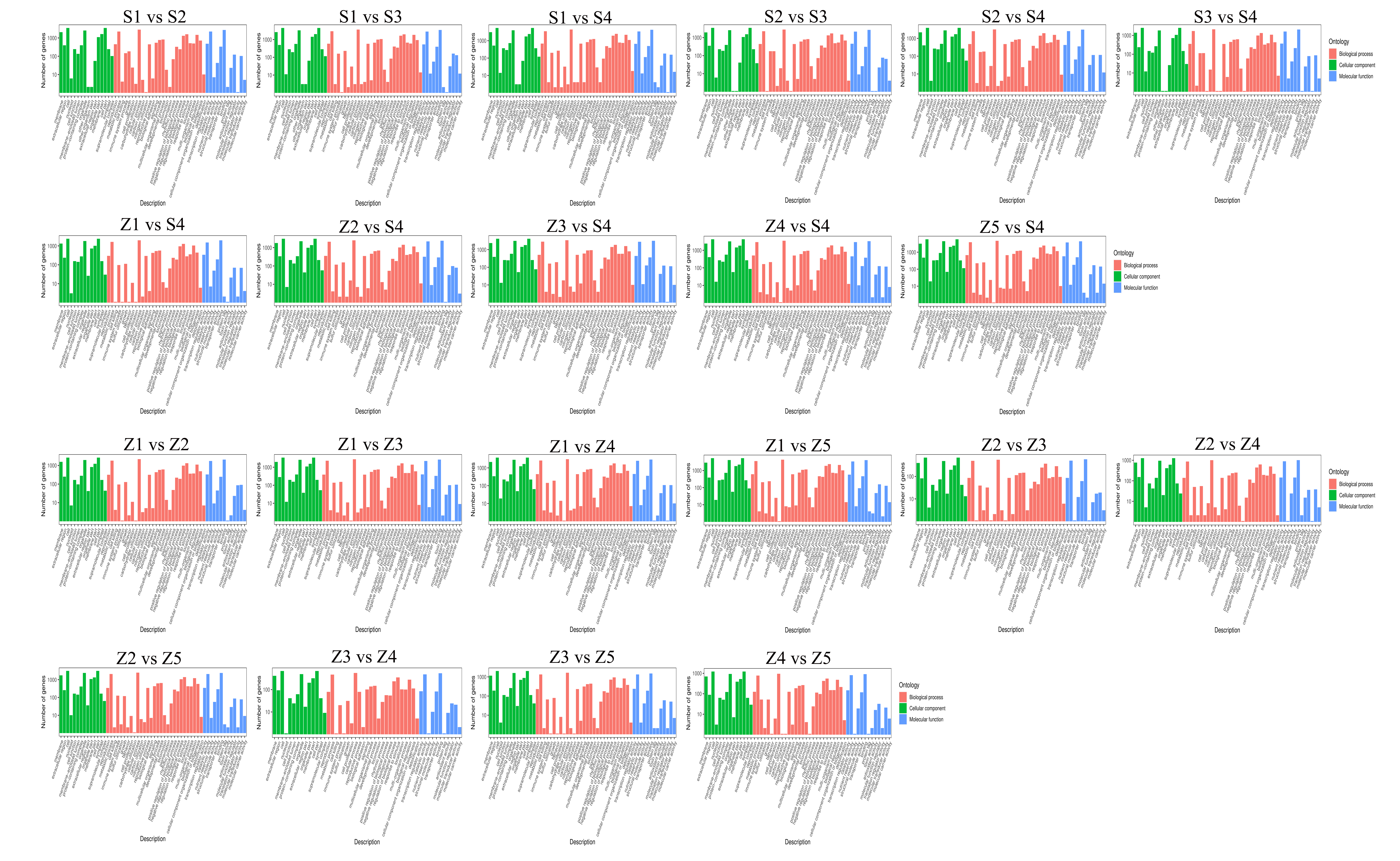

Supplement: Supplementary file 1 [file life-12-01494-s001.zip › Figure S2.tif]

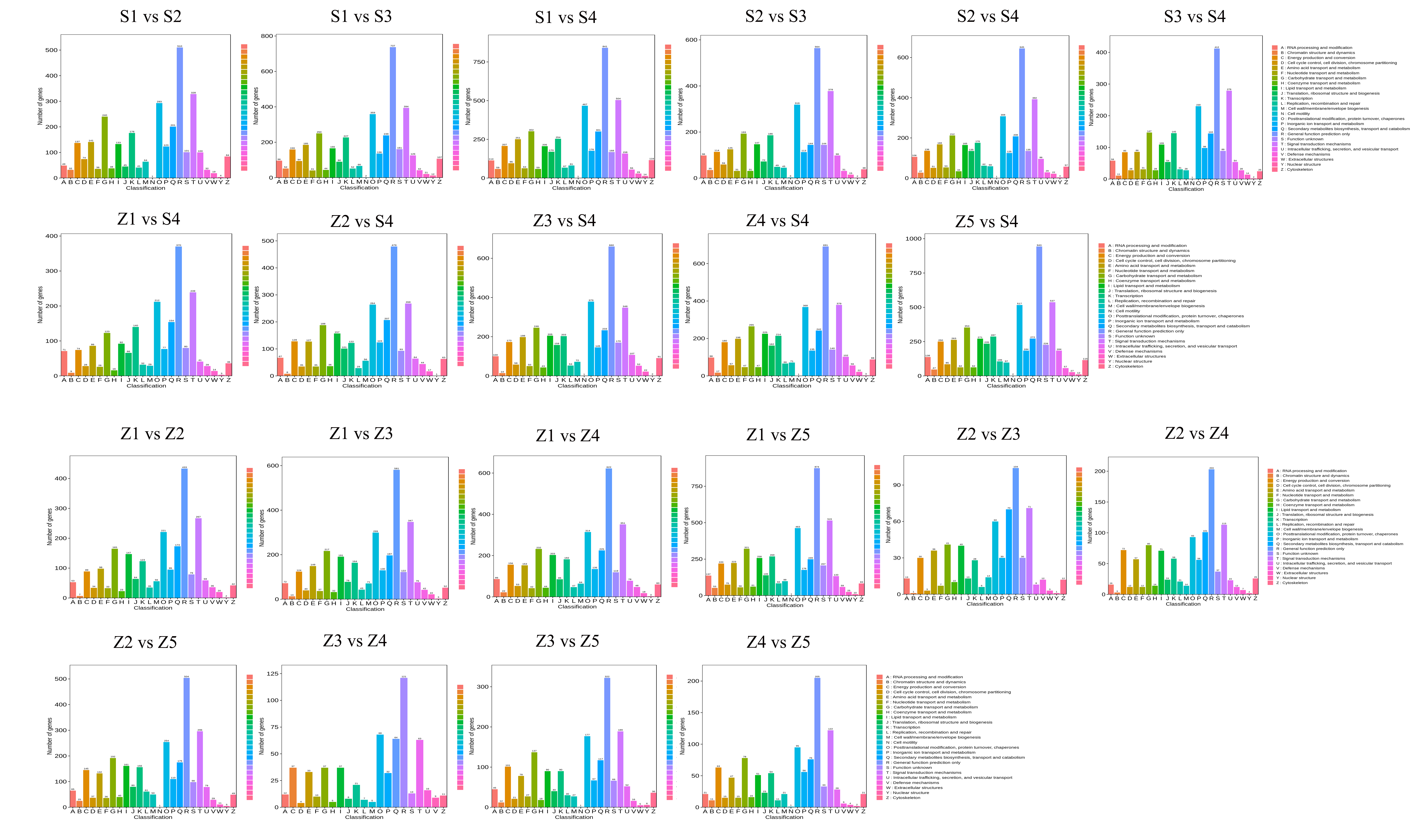

Supplement: Supplementary file 1 [file life-12-01494-s001.zip › Figure S3.tif]

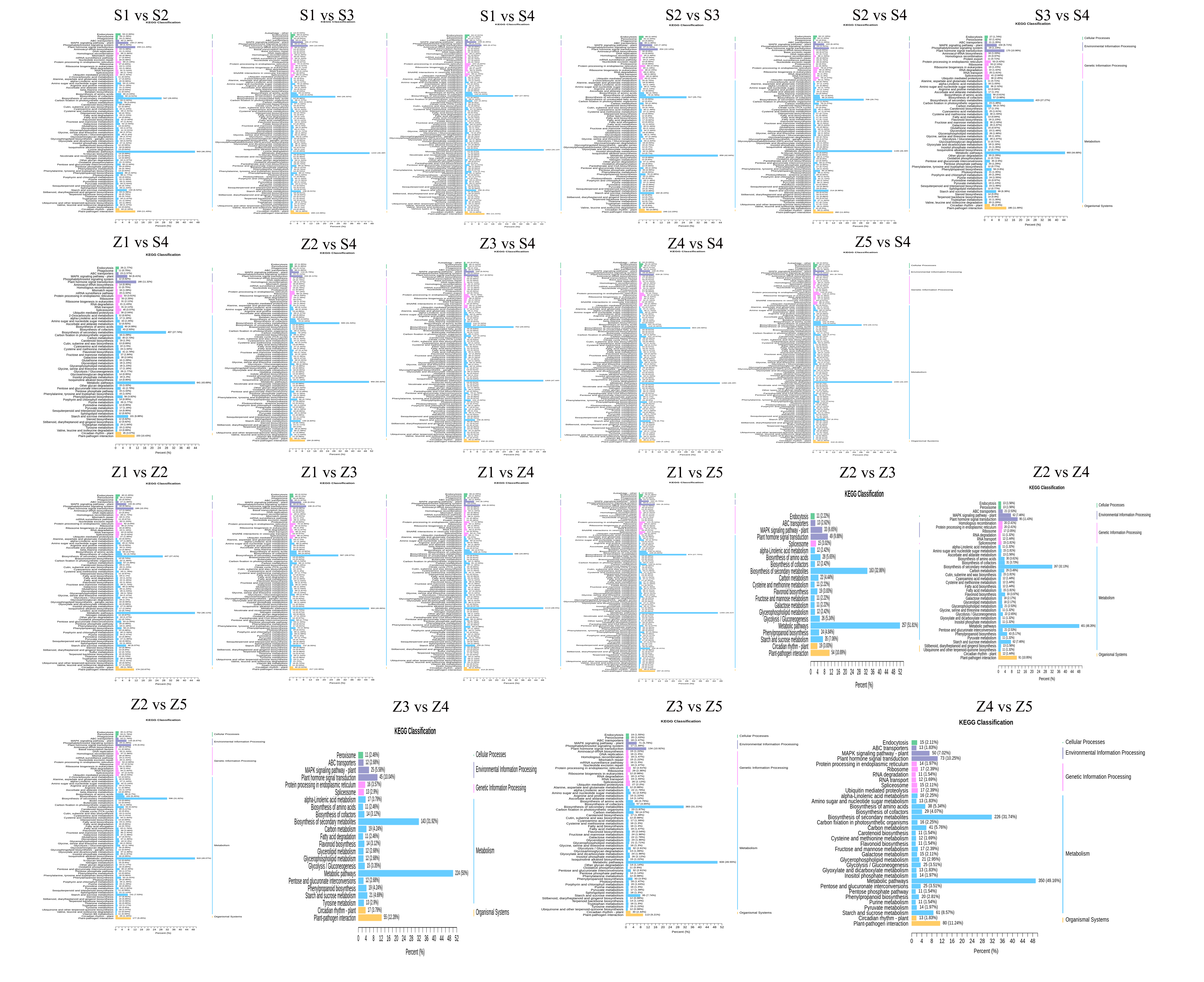

Supplement: Supplementary file 1 [file life-12-01494-s001.zip › Figure S4.tif]

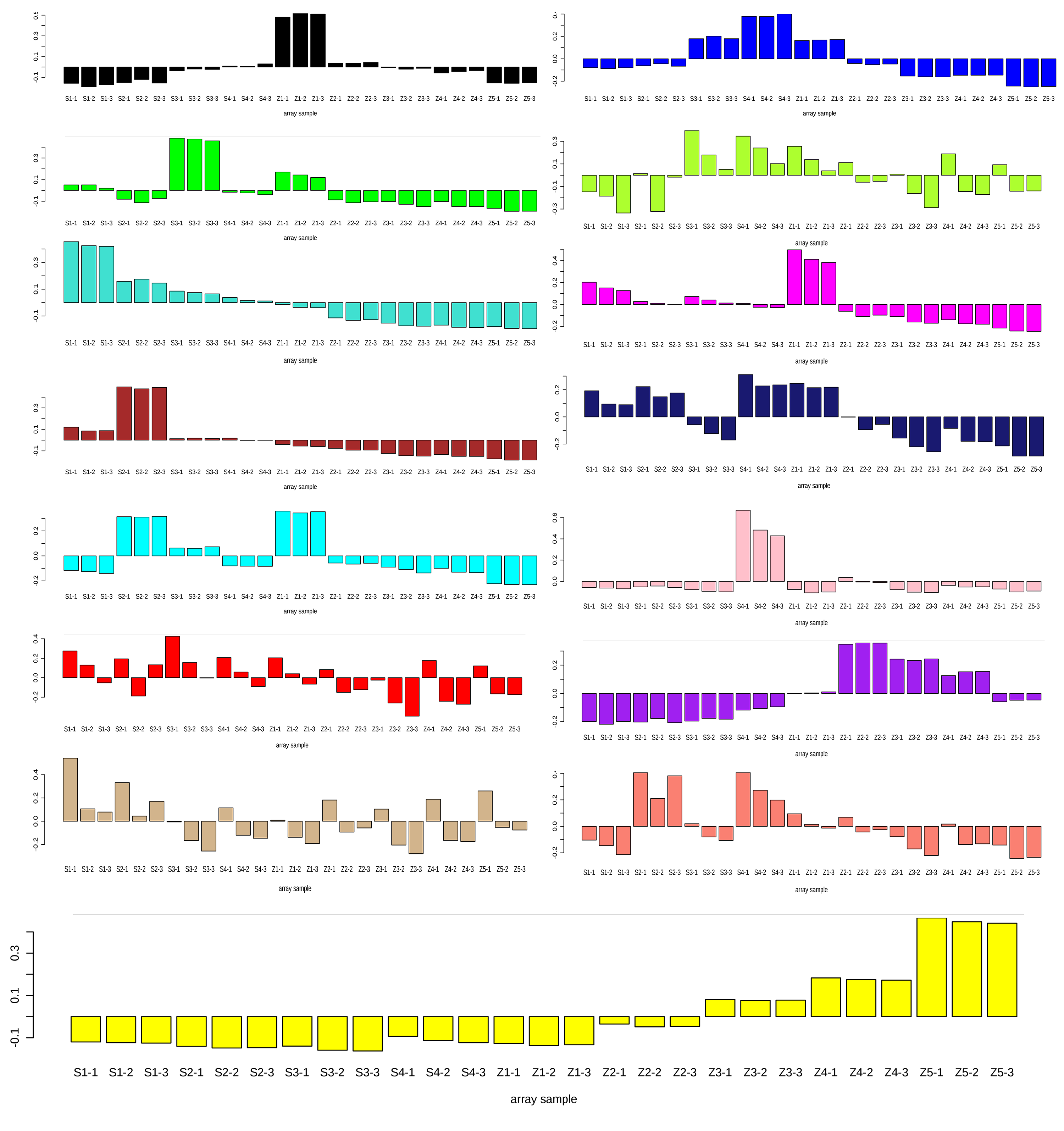

Supplement: Supplementary file 1 [file life-12-01494-s001.zip › Figure S5.tif]
